# Supplementary material for: IL-33-mediated mast cell activation promotes gastric cancer through macrophage mobilization
Source: Nat Commun. 2019 Jun 21;10:2735. doi: 10.1038/s41467-019-10676-1 (PMC6588585; doi:10.1038/s41467-019-10676-1)
Supplement: Supplementary file 1 — Supplementary Information [file 41467_2019_10676_MOESM1_ESM.pdf]

Eissmann et al. Supplementary Info and Data

“IL-33-mediated mast cell activation promotes gastric cancer through macrophage mobilization.”

Supplementary Methods

Peripheral blood composition analysis

Heart bleeds were performed on *gp130FF;c-Kit*$^{+/+}$ and *gp130FF;c-Kit*$^{W\text{-}sh/W\text{-}sh}$ mice, blood was collected into EDTA K coated-collection tubes (Sarstedt) and analyzed with an ADVIA 2120 hematology analyzer (Siemens).

Bone marrow transfer

6 week old mice were lethally radiated and reconstituted with bone marrow cells. Bone marrow chimera of gp130^{++} -> *gp130*^{FF} (wt-> FF) , *gp130*^{FF} -> *gp130*^{FF} (FF-> FF), mice were generated. Eight weeks after reconstitution of the bone marrow mice were euthanized and subsequently analyzed.

Protein extraction and immunoblotting

Whole cell protein lysates were prepared in RIPA lysis buffer supplemented with protease and phosphatase inhibitor tablets (Roche) with a TissueLyser II (Qiagen). Protein concentration was determined by the bicinchoninic acid (BCA) assay. Then, tissue lysates were reconstituted in 4x Laemmli's loading buffer, separated via sodium dodecyl sulphate polyacrylamide gel electrophoresis and were dry-transferred to nitrocellulose membranes using a iBlot (Invitrogen). Membranes were incubated overnight at 4°C with the primary antibodies goat anti-mouse IL-33 (1/500 dilution, R&D Systems, Cat# AF3626, RRID:AB_884269) and rabbit anti-mouse Gapdh (1/5000 dilution, Sigma-Aldrich, Cat# G9545, RRID:AB_796208) followed by 1 hour incubation with fluorescent conjugated secondary antibodies IRDye 680 donkey anti-goat (1/10000 dilution, LI-COR Biosciences, Cat# 926-68074, RRID:AB_10956736) and IRDye 800 goat anti-rabbit (1/10000 dilution, LI-COR Biosciences, Cat# 926-32211, RRID:AB_621843).

Proteins were visualized using the Odyssey Infrared Imaging System (LICOR Bio-sciences).

Macrophage migration assay

A modified Boyden-chamber transwell assay was performed. Bone marrow derived macrophages (BMDM) were seeded at $2x10^5$ cells / transwell insert (cell culture Inserts 8.0 µm Translucent PET membrane for 24-well plates, FAL353097, In Vitro Technologies). Chemotactic media were in the bottom, collection well. Recombinant mouse proteins Mcp1, Mip-1α, Mip-1β, Mcp3 and Gm-csf (all Biolegend) were tested at 30 ng/ml concentration. After incubated for 6 h cells on the top of the membrane were removed. After counterstaining, cells on the bottom side of the membrane were counted under a microscope. Migratory potential was calculated as fold change of test medium to negative control.

Stat3 Chromatin Immunoprecipitation-Sequencing (ChIP-Seq) analysis

Gp130^{FF} mice were administered 5µg of recombinant IL-11 or vehicle (PBS; four mice each) via peritoneal injection and 60 min later mice were euthanatized, stomach tumors dissected, processed and DNA-Protein cross-linking was performed using formaldehyde. After tissue disaggregation and homogenization using the Medimachine tissue grinder equipped with a 50 µl Medicon (Becton Dickinson), cell lysis, nuclei extraction and nuclei lysis were performed. Chromatin shearing was carried out by sonification with the Bioruptor (Diagenode). The chromatin immunoprecipitation (ChIP) reaction was then performed using the Imprint ChIP Kit (Sigma) according to manufacturer's instructions and a ChIP-grade Stat3 antibody (Santa Cruz #482X). After DNA elution, cross-link reversal, purification (QIAquick PCR Purification Kit; Qiagen) and quantification (Quant-iT PicoGreen dsDNA Kit; Invitrogen), two ChIP DNA samples ('untreated' and 'IL11' with pooled ChIP DNA from four mice each) were submitted to whole genome sequencing (35 bp, single end) by Geneworks (Australia) using the Genome Analyzer II (Illumina). Sequencing raw data of the fastq file format

(GSE48285) was aligned to the reference genome (M_musculus_Jul_2007) with Bowtie software and peak calling – identification of significantly enriched regions – was performed with the Model-based Analysis of ChIP-Seq (MACS) software using a fold-enrichment ratio of 12-fold. Finally, peak lengths, locations and associated genes were identified using R scripts and peaks were visualized using the Integrated Genome Browser (IGB) software. The DNA sequence of the *Il33* gene-associated Stat3-binding peak was scanned for consensus Stat3-binding motif (MOTIFMAP ID: M00225) with MAST software (http://meme-suite.org). ChIP-Seq raw and alignment data as well as MACS output data are publicly available at NCBI gene expression omnibus (GEO) using the accession number GSE48285 [https://www.ncbi.nlm.nih.gov/geo/query/acc.cgi?acc=GSE48285].

Quantitative RT-PCR

Quantitative RT-PCR analyses were performed in technical triplicates with SensiMix SYBR kit (Bioline, Cat# QT605-20)) using the *ViiA*™ *7* Real Time *PCR* System (life technologies over 40 cycles (94ºC/20 s, 60ºC/15 s, 72ºC/15 s), following an initial denaturation step at 95ºC/10 min. The housekeeping gene *Gapdh* was used to normalize cDNA concentrations of target genes. Fold changes in gene expression were obtained using the 2-ddCT method. The oligonucleotides used are listed in Table S1.

Supplementary Figures

Supplementary Figure 1

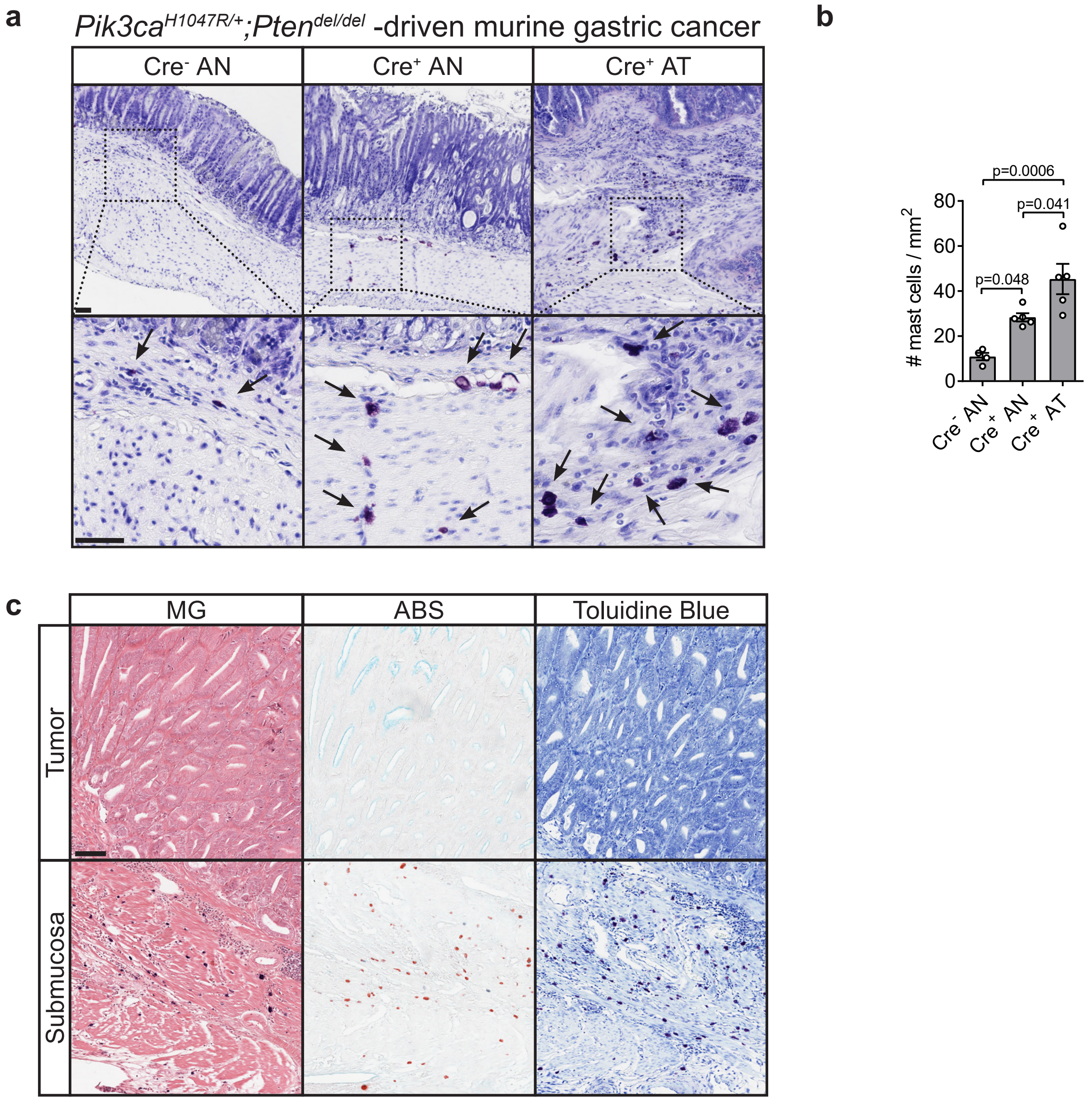

Fig. S1 Mast cells in *Pik3ca*$^{H1047R/+}$;*Pten*$^{fl/fl}$ –driven gastric tumors (related to Fig. 1)

a Representative cross-section of Toluidine Blue-stained stomach comprising either antrum (AN) or antral tumors (AT) of Tg*(Tff1-CreERT2);Pik3ca*$^{H1047R/+}$;*Pten*$^{fl/fl}$ mice six month post Tamoxifen-mediated activation of the mutant alleles. Mast cells (arrow) appear purple. Scale bars = 50 µm.

b Quantification of submucosal mast cell in sections depicted in **A**. n = 4 mice (*Cre*$^{-}$ AN) and n = 5 (*Cre*$^{+}$ AN and *Cre*$^{+}$ AT). One-way ANOVA was performed with F (DFn, Dfd) = 14.56 (2, 11).

c Representative consecutive sections of gastric submucosa and tumors of *gp130*$^{F/F}$ mice, and stained with May-Grunwald-Giemsa (MG), Alcain blue/Safranin O (ABS) and Toluidine Blue for detection of mast cells. Scale bars = 100 µm.

Data are represented as mean ± SEM, with p values $p < 0.05$ considered being significant. Source data are provided as a Source Data file.

Supplementary Figure 2

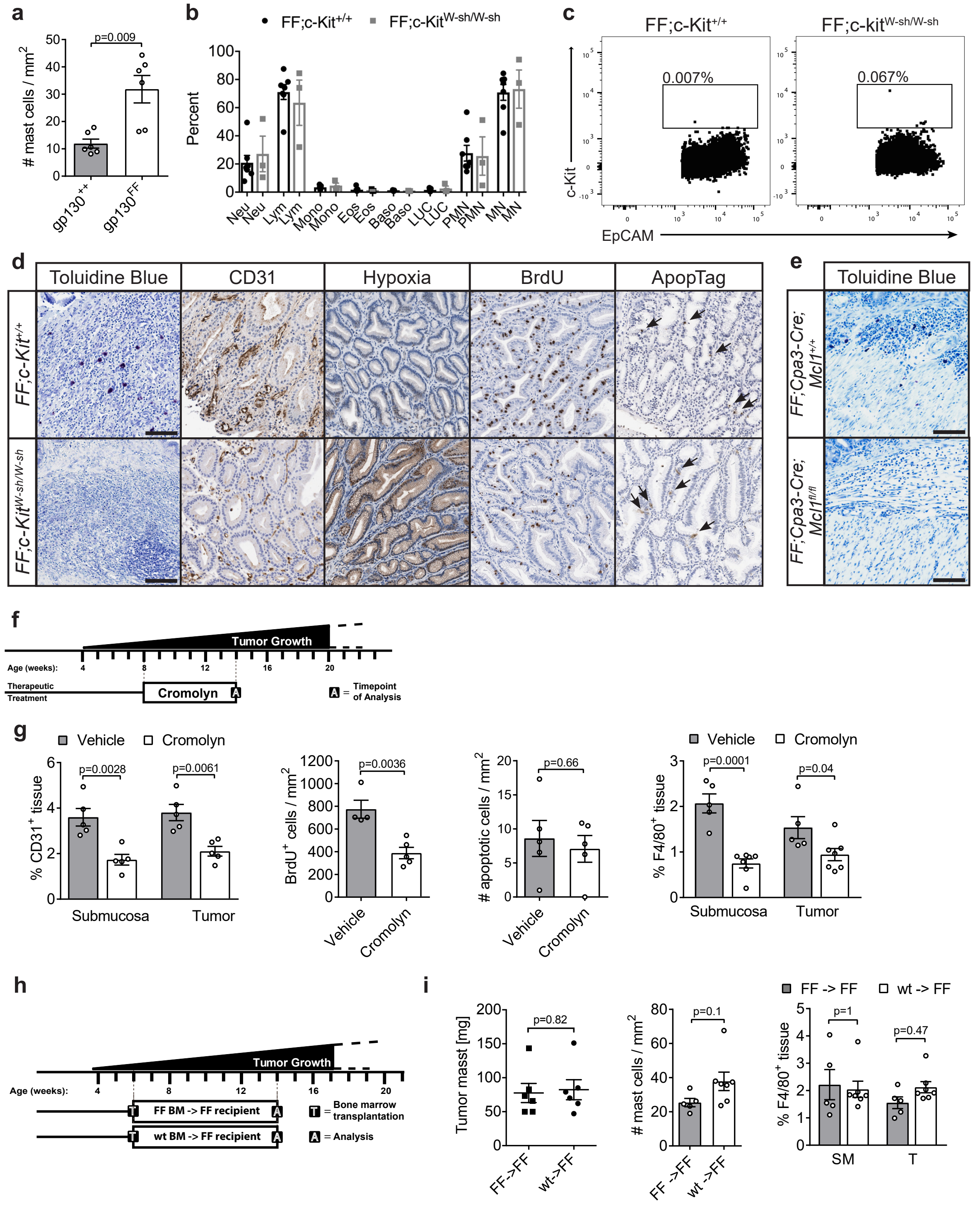

Fig. S2 Mast cell-dependent gastric tumor growth (related to Fig. 2)

a Submucosal mast cells frequency on Toluidine Blue-stained stomach sections from 4-week-old wild-type (*gp130*$^{++}$) and *gp130*^{FF} mice prior to onset of tumorigenesis. n = 6, from two independent experiments with t-test t (df) = 3.78 (6.06).

b Cell composition of heart bleed samples from gp130^{FF};c-Kit$^{+/+}$ (FF;c-Kit$^{+/+}$) and gp130^{FF};c-Kit$^{W\text{-}sh/W\text{-}sh}$ (FF;c-Kit$^{W\text{-}sh/W\text{-}sh}$) mice at 100 day of age. n = 4 (*FF;c-Kit*$^{+/+}$) and n = 3 (*FF;c-Kit*$^{W\text{-}sh/W\text{-}sh}$).

c Flow cytometric c-Kit expression of gastric tumor-derived EpCam^{+} cells from *gp130*^{FF} mice. Dot plot for *gp130*^{FF}*;c-Kit*$^{W\text{-}sh/W\text{-}sh}$ –derived tumor epithelial cells as control.

d Representative images for submucosal mast cells (Toluidine Blue), and tumor sections for macrophages (F4/80), angiogenic vessels (CD31), proliferation (BrdU), hypoxia (Hypoxy-probe stain) and apoptosis (ApopTag) in 100 day old mice *gp130*^{FF} mice. Scale bars = 100 µm.

e Toluidine blue stained stomach sections of indicated genotype. Scale bars = 100 µm.

f Illustration of tumourigenesis kinetics in *gp130*^{FF} and the treatment regiment.

g Quantification of *gp130*^{FF} tumor sections of mice treated with either vehicle (PBS) or cromolyn assessing angiogenic vessels (CD31), proliferation (BrdU), apoptosis (ApopTag) and macrophages (F4/80). CD31: n = 5, one-way ANOVA F (DFn, Dfd) = 11.58 (3, 16); BrdU: n = 4-5, t-test t (df) = 4.3 (7); apoptotic cells: n = 5, t-test t (df) = 0.463 (8); F4/80: n = 5-7, one-way ANOVA F (DFn, Dfd) = 12.87 (3, 20).

h Graphic of adaptive bone marrow transfer between *gp130*^{FF} recipient mice (*FF*) and wild type (*wt)* or FF bone marrow (BM).

i Tumor burden of bone marrow chimeras as in **g** and quantification of Toluidine Blue-positive submucosal mast cells and F4/80^{+} macrophages associated with submucosa (SM) and tumors (T) in bone marrow chimeras. Tumor mass: n = 5 mice, . t-test t (df) = 0.24 (10); mast cells: n = 5-7, t-test t (df) = 1.83 (10); F4/80: n = 5-7, one-way ANOVA F (DFn, Dfd) = 0.72 (3, 20).

Data are represented as mean ± SEM, with p values $p < 0.05$ considered being significant. Source data are provided as a Source Data file.

Supplementary Figure 3

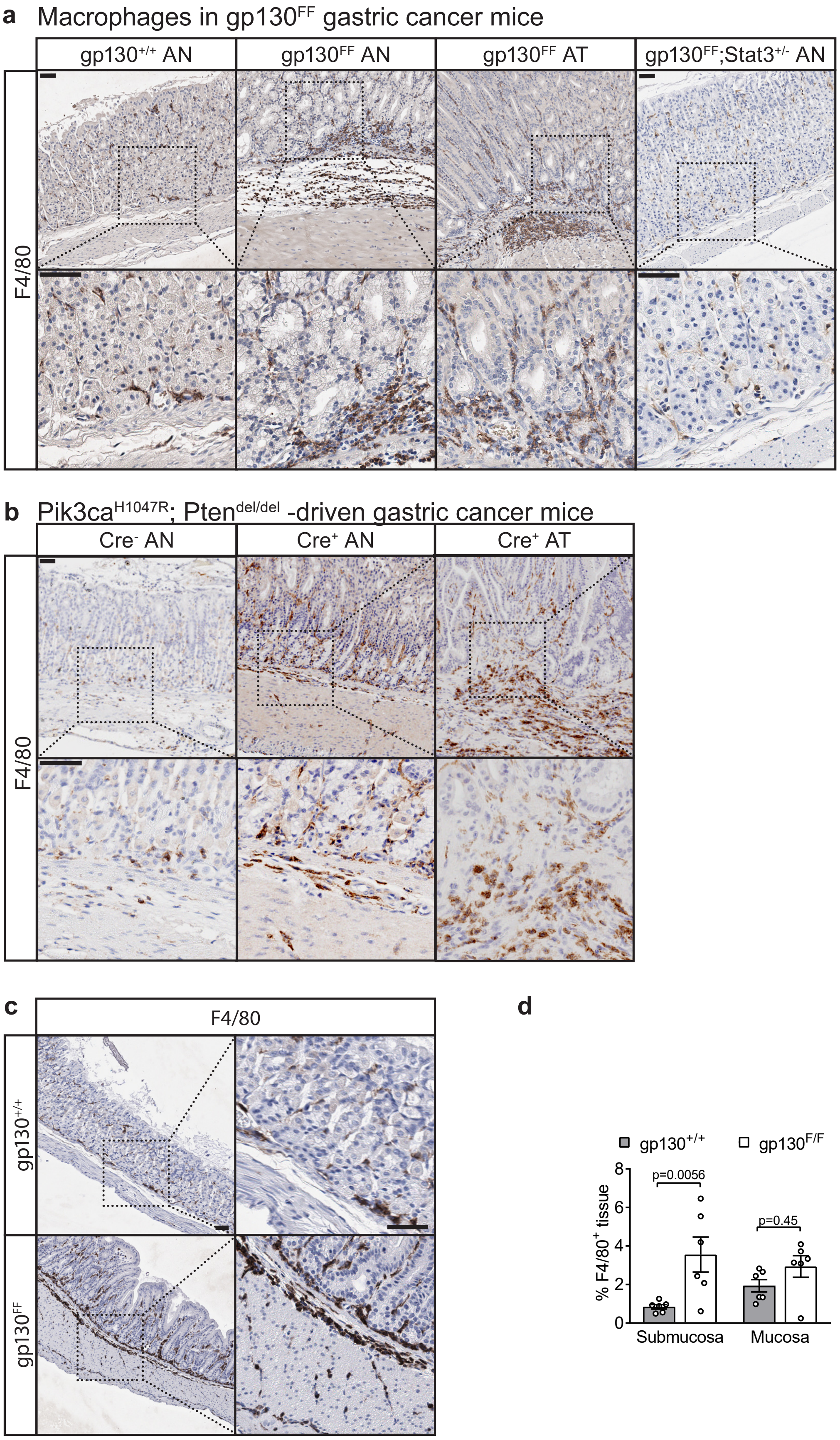

Fig. S3 Macrophage density during gastric tumorigenesis in mice (related to Fig. 3)

a Representative immunohistochemical stainings for F4/80-positive macrophages associated in unaffected antrum (AN) or antral tumors (AT) in 100 day old mice of the indicated genotype. Scale bars = 50 µm.

b Representative immunohistochemical stainings for F4/80-positive macrophages associated with unaffected antrum (AN) or antral tumors (AT) in mice (180 days post mutant allele induction) from the Tg(*Tff1-CreERT2)*;*Pik3ca*$^{H1047R/+}$;*Pten*$^{fl/fl}$ strain that either harbor (Cre^{+}) or lack (Cre^{-}) the *Tff1-CreERT2* driver allele. Scale bars = 50 µm.

c Representative images of sections stained for F4/80-positive macrophages in 4 week old mice of the indicated genotype. Scale bars = 50 µm.

d Quantification of F4/80 positive macrophages of mice as in **c**. n = 6 mice; one-way ANOVA F (DFn, Dfd) = 4.46 (3, 20).

Data are represented as mean ± SEM, with p values $p < 0.05$ considered being significant. Source data are provided as a Source Data file.

Supplementary Figure 4

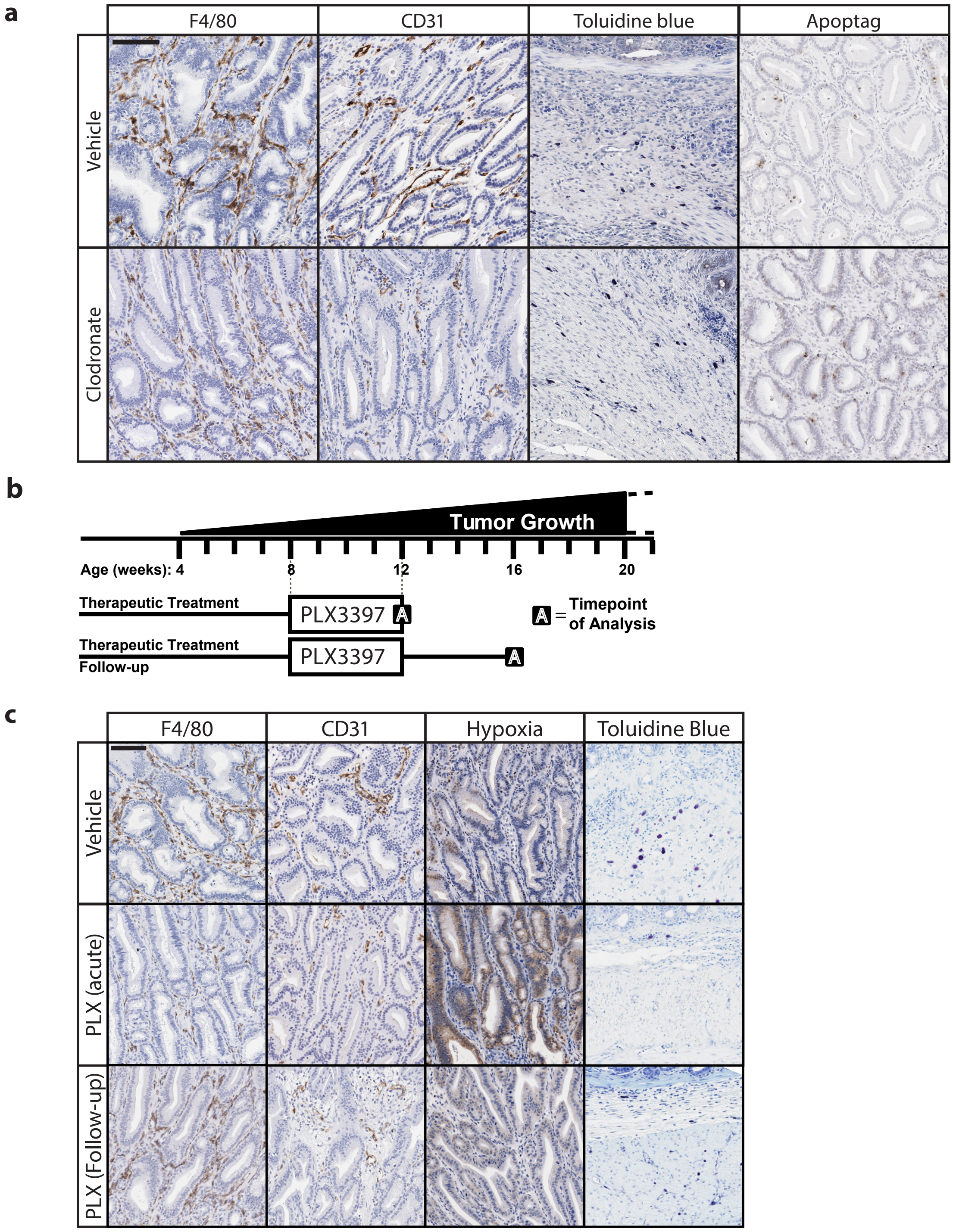

Fig. S4 Pharmacological depletion of macrophages in *gp130*^{FF} gastric tumors (related to Fig. 4 & 5)

a Representative immunohistochemical stainings for tumor-adjacent submucosal mast cells (Toluidine Blue), and in tumor sections for macrophages (F4/80), angiogenic vessels (CD31) and

apoptosis (ApopTag) of *gp130*^{FF} mice at the end of a 6 week treatment with vehicle or clodronate-treated mice when the mice had reached 100 days of age. Scale bars = 100 µm.

b Schematic illustration of the kinetics of tumorigenesis in *gp130*^{FF} and treatment/drug-free follow-up regime using PLX3397 (Csf1r/C-kit/Flt3 tyrosine kinase receptor inhibitor).

c Representative immunohistochemical stainings for tumor-adjacent submucosal mast cells (Toluidine Blue), and in tumor sections for macrophages (F4/80), angiogenic vessels (CD31) and hypoxia (Hypoxy-probe stain) of *gp130*^{FF} mice at the end of a treatment period as outlined in **b.** Scale bars = 100 µm.

Supplementary Figure 5

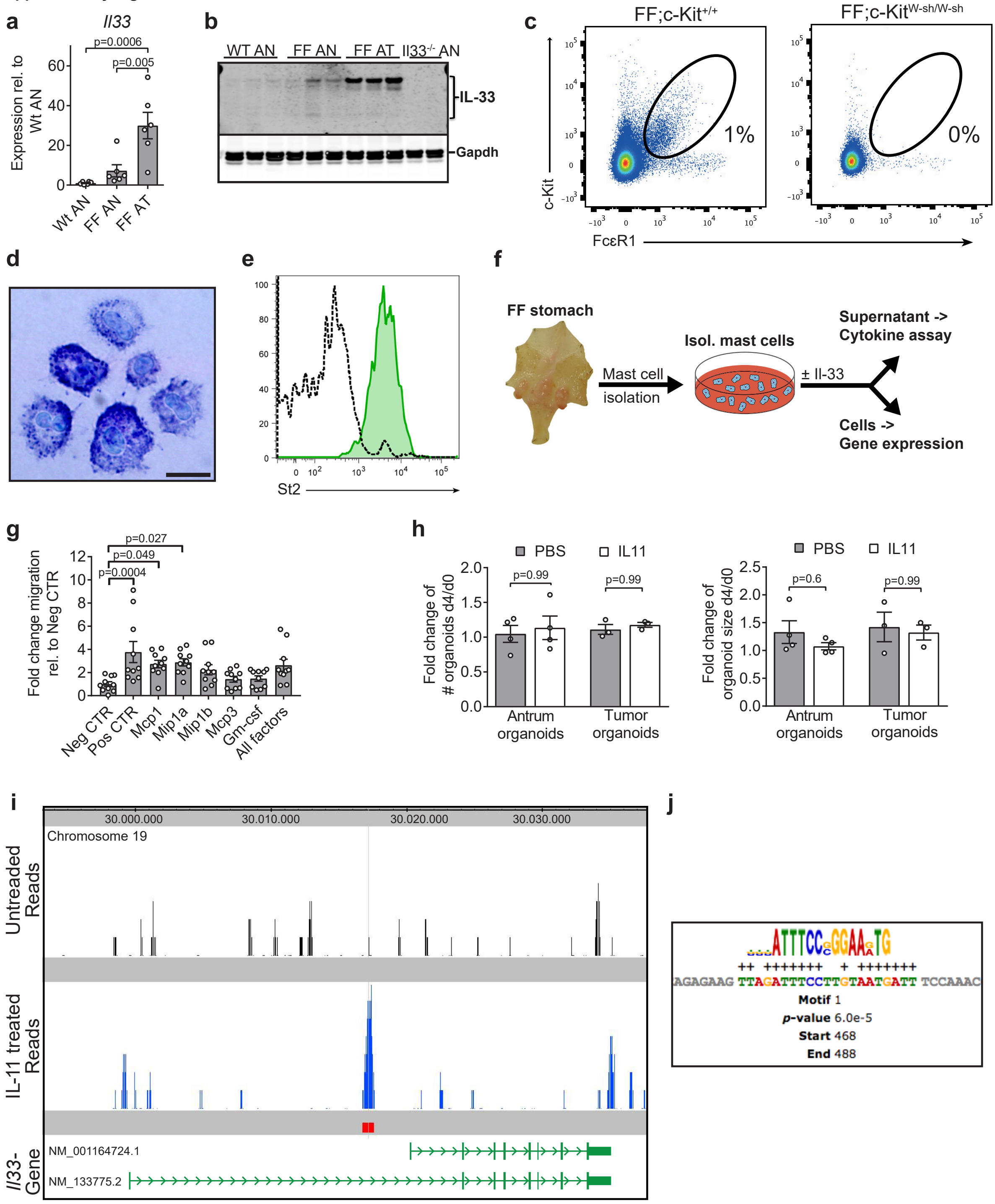

Fig. S5 IL-33 expression in gastric cancer and IL-33 stimulation of mast cells (related to Fig. 6)

a *Il33* mRNA expression analysis in unaffected antrum (AN) and antral tumors (AT) of 100 day old wild-type (WT) and $gp130^{FF}$ (FF) mice. Data are normalized to *Gapdh* and plotted as relative

expression to WT AN expression n = 6 mice, from two independent experiments. One-way ANOVA was performed with F (DFn, Dfd) = 12.93 (2, 15).

b IL-33 protein immunoblotting of whole tissue lysates of antrum (AN) from wild-type (WT) and *gp30*^{FF} mice (FF), and of *gp130*^{FF} tumor (FF AT). Antrum from *IL33*$^{-/-}$ mice served as a specificity control. Each lane represents an individual mouse.

c Flow cytometric analysis for c-Kit and FcεR1 expression of CD45^{+}, EpCAM^{-}, CD11b^{-} cells isolated from *gp130*^{FF} and mast cell-deficient *gp130*^{FF}*;c-Kit*$^{W\text{-}sh/W\text{-}sh}$ stomachs.

d Representative Toluidine Blue staining of cyto-spun FACS-sorted c-Kit^{+};FcεR1^{+} stomach mast cells as in **c**. Scale bar = 10 µm

e Flow cytometric analysis of St2 expression on FACS-sorted ckit^{+};FcεR1^{+} stomach mast cells as described in **c**, and derived from *gp130*^{FF} mice (green) or *gp130*^{FF}*;St2*$^{-/-}$ mast cells (dotted black line).

f Schematic illustration of mast cell isolation and in vitro stimulation experiment with IL-33 (30 ng/ml) for 3 h. Supernatants were analyzed via multiplex cytokine assay and cell pellets were processed for gene expression analysis via qPCR.

g Migration assay was performed with *gp130*^{FF}-mutant bone marrow derived macrophages testing the chemo-attracting capacity of the depicted factors. N = 10, from four independent experiments. Neg. CTR = FCS-free medium, Pos. CTR= 10% FCS medium, all single factors we supplied at 30 ng/ml in Neg CTR medium. One-way ANOVA was performed with F (DFn, Dfd) = 4.1 (7, 72).

h Gastric organoids from either the antrum or from the tumor of *gp130*^{FF} mice were treated with 100 ng/ml recombinant IL-11 for 4 days and organoid numbers and organoids size were determined for that period. n = 4 (antrum) and n = 3 (tumor) mice derived independent organoids. Fold change #: One-way ANOVA F (DFn, Dfd) = 0.19 (3, 10), fold change size: One-way ANOVA F (DFn, Dfd) = 0.75 (3, 10).

i Stat3-Chip-Seq experiment was performed with tumor tissue from either untreated (vehicle) or IL-11 treated *gp130*^{FF} mice (n = 4 mice each). Stat3-associated peaks at the *Il33* gene locus are illustrated with the Integrated Genome Browser software. The red horizontal bar(s) below the peaks indicate statistically significant Stat3 recruitment calculated using the MACS software.

j The DNA-sequence associated with the significant peak in the IL-11 sample (chr19:30016764-30017654; shown in **i**) was scanned for consensus Stat3 binding motifs (MOTIFMAP ID: M00225) using MAST online software. The upper sequence represents the Stat3 binding motif and aligned below a part of the peak sequence within the *Il33* gene locus is shown.

Data are represented as mean ± SEM, with p values $p < 0.05$ considered being significant. Source data are provided as a Source Data file.

Supplementary Figure 6

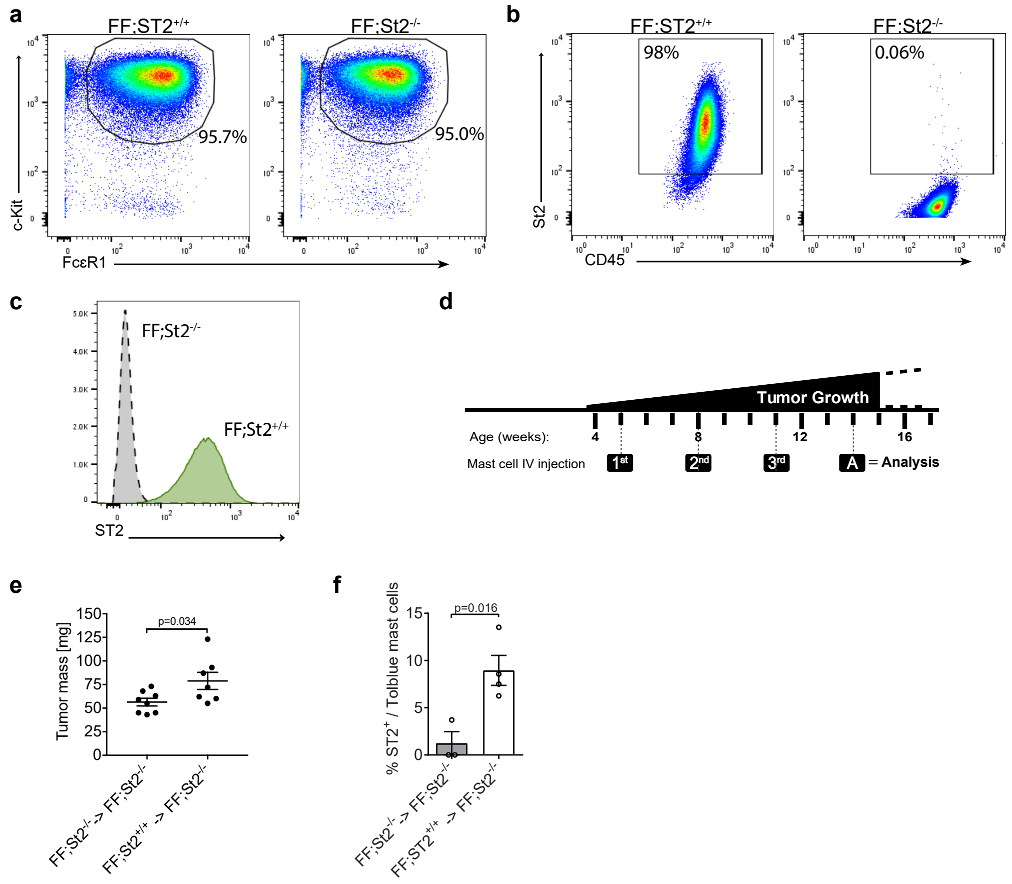

Fig. S6 Adaptive transfer of St2$^{+/+}$ mast cells into *gp130^{FF};ST2$^{-/-}$* host (related to Fig. 7)

a–**c** Flow cytometric analysis of *in vitro* amplified BMMCs of indicated genotype. After pre-gating for CD45^{+}, CD11b^{-} cells, the percentage of c-Kit^{+},FceR1^{+} mast cells was determined (**a**) and the frequency of St2 expression within the c-Kit^{+},FceR1^{+} mast cell populations is shown as dot blots (**b**) and in an overlay histogram (**c**.)

d An graphic illustrates the timeline of the BMMC transplantation experiment. At mouse age 10^{7} BMMC of either *FF,St2$^{+/+}$* or *FF,St2$^{-/-}$* genotype were injected via tail vine into *FF,St2$^{-/-}$* recipient mice. Tumor analysis occurred three weeks post last BMMC injection at 14 weeks of age.

e Alternative analysis of the tumor mass data of the BMMC adaptive transfer experiment shown in Fig. 6g. Here, one data point (249mg tumor burden, *FF,St2$^{-/-}$* -> *FF,St2$^{-/-}$*) was removed from the data set after ROUT (Q=0.1%) outlier analysis and unpaired t-test was performed (t (df) = 2.369 (13)).

f Consecutive stomach sections from BMMC transplantation experiment were stained for St2 and Toluidine blue. The number of $ST2^+$ cells and Tolblue mast cells in the submucosa was quantified. The graph represents % of mast cells being St2-positive as calculated by 100*(# $St2^+$ cells / # $Tolblue^+$ cells). N= 3 (*FF,St2*$^{-/-}$ -> *FF,St2*$^{-/-}$) and n = 4 (*FF,St2*$^{+/+}$ -> *FF,St2*$^{-/-}$) mice were analyzed (unpaired T-test t(df) = 3.6 (5)).

Data are represented as mean ± SEM, with p values $p < 0.05$ considered being significant. Source data are provided as a Source Data file.

Supplementary Figure 7

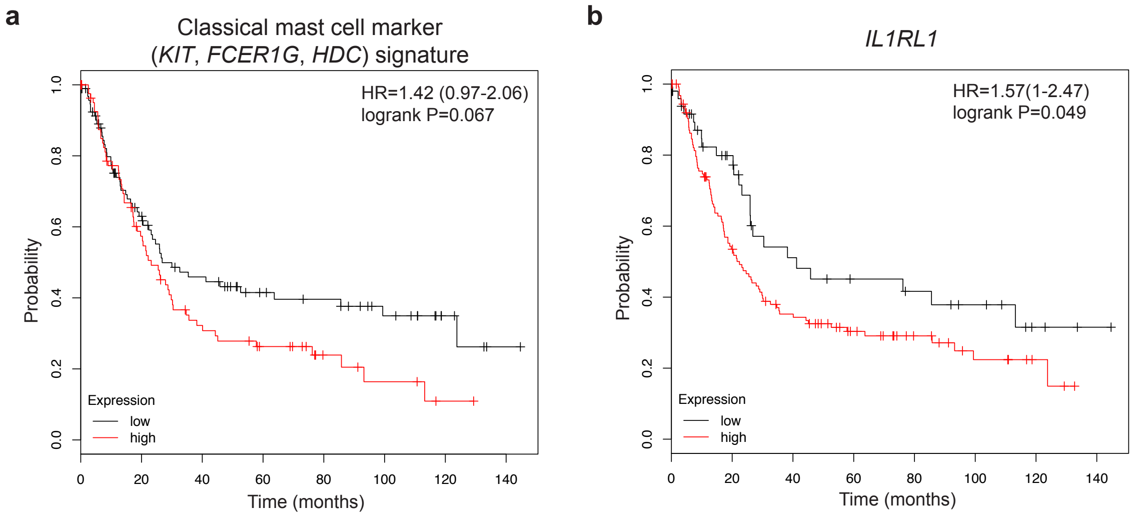

Fig. S7 Kaplan-Meyer analysis of mast cell markers and IL1RL1 expression (related to Fig. 8)

a, b Kaplan-Meyer Survival analysis was conducted for a classical mast cell marker gene expression signature comprised of KIT, FCER1G and HDC (**a**) and St2-receptor encoding IL1RL1 gene (**b**) in intestinal-type gastric cancer patients.

Supplementary Figure 8

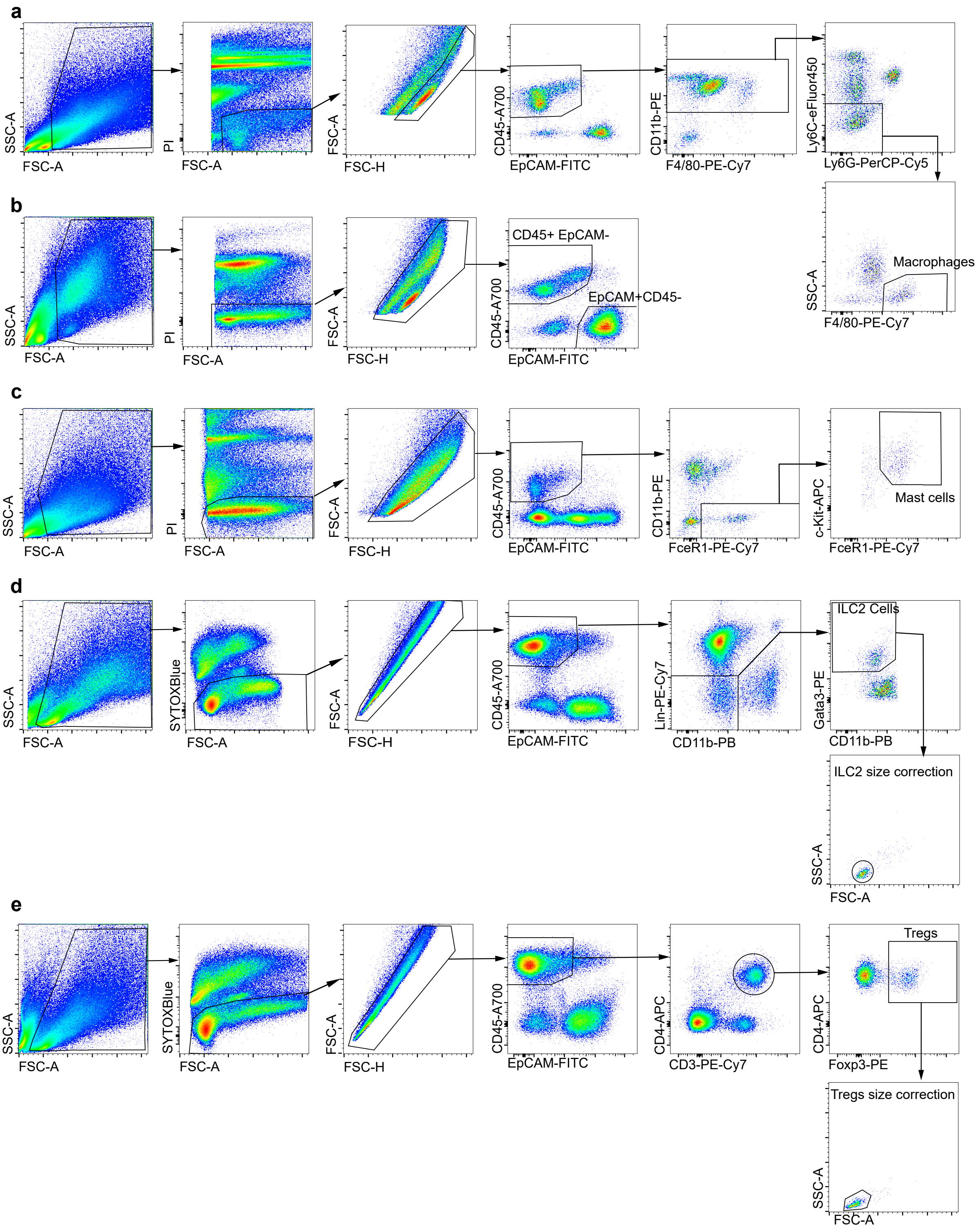

Fig. S8 Gating strategies used for cell sorting and flow cytometric analysis

a Gating strategy used to sort macrophages from mouse stomachs for expression analysis presented on Fig. 5c.

b $CD45^{+}EpCAM^{-}$ and $EpCAM^{+}CD45^{-}$ cell populations were sorted with shown gating strategy. Cells were used for gene expression analysis presented on Fig 6a.

c Gating performed to sort gastric mast cells for IL-33 stimulation presented on Fig 6c,d and Fig. 7d.

d Gating strategy conducted to quantify ILC2 frequency presented on Fig. 7e.

e Gating conducted for Treg cell frequency analysis presented on Fig. 7f.

Supplementary Tables

Supplementary Table 1   Oligonucleotide sequences for quantitative RT-PCR

Gene	Forward 5-3’	Reverse 5-3’
Il33	TCCAACTCCAAGATTTCCCCG	CATGCAGTAGACATGGCAGAA
Il1rl1 *	TCTGCCCGACGTTCTTGAAA	CATGTGAGGGGCCAGAACAA
Socs3	GCGGGCACCTTTCTTATCC	TCCCCGACTGGGTCTTGAC
Arg1	AGGACAGCCTCGAGGAGGGG	TGGACCTCTGCCACCACACCAG
Fizz1	CCCAGGATGCCAACTTTGAA	GGCCCATCTGTTCATAGTCT
Mrc1	TTTGGAATCAAGGGCACAGAG	TGCTCCACAATCCCGAACC
Nos2	GCCACCAACAATGGCAACA	CGTACCGGATGAGCTGTGAATT
Vegfa	CCAGGCTGCACCCACGACAG	CGGCACACAGGACGGCTTGA
Ccl2	CATCCACGTGTTGGCTCA	GCTGCTGGTGATCCTCTTG
Ccl3	TTCTCTGTACCATGACACTCTGC	CGTGGAATCTTCCGGCTGTAG
Ccl7	TTCTGTGCCTGCTGCTCATA	TTGACATAGCAGCATGTGGAT
Csf2	CTCACCCATCACTGTCACCC	TCTTCATTCAACGTGACAGGC
Il6	TCTATACCACTTCACAAGTCGGA	GAATTGCCATTGCACAACTCTTT

Displayed are the gene names and the corresponding forward and reverse Primer sequences used for quantitative RT-PCR analysis. All Primer pairs are detecting the mouse genes. * indicates the membrane bound isoform.
