# Supplementary material for: IL-33-mediated mast cell activation promotes gastric cancer through macrophage mobilization
Source: Nat Commun. 2019 Jun 21;10:2735. doi: 10.1038/s41467-019-10676-1 (PMC6588585; doi:10.1038/s41467-019-10676-1)
Supplement: Supplementary file 3 — Reporting Summary [file 41467_2019_10676_MOESM3_ESM.pdf]

nature research

Corresponding author(s): Matthias Ernst

Last updated by author(s): May 6, 2019

# Reporting Summary

Nature Research wishes to improve the reproducibility of the work that we publish. This form provides structure for consistency and transparency in reporting. For further information on Nature Research policies, see Authors & Referees and the Editorial Policy Checklist.

## Statistics

For all statistical analyses, confirm that the following items are present in the figure legend, table legend, main text, or Methods section.

| n/a | Confirmed | |
|---|---|---|
| ☐ | ☒ | The exact sample size (*n*) for each experimental group/condition, given as a discrete number and unit of measurement |
| ☐ | ☒ | A statement on whether measurements were taken from distinct samples or whether the same sample was measured repeatedly |
| ☐ | ☒ | The statistical test(s) used AND whether they are one- or two-sided<br>*Only common tests should be described solely by name; describe more complex techniques in the Methods section.* |
| ☒ | ☐ | A description of all covariates tested |
| ☐ | ☒ | A description of any assumptions or corrections, such as tests of normality and adjustment for multiple comparisons |
| ☐ | ☒ | A full description of the statistical parameters including central tendency (e.g. means) or other basic estimates (e.g. regression coefficient) AND variation (e.g. standard deviation) or associated estimates of uncertainty (e.g. confidence intervals) |
| ☐ | ☒ | For null hypothesis testing, the test statistic (e.g. *F*, *t*, *r*) with confidence intervals, effect sizes, degrees of freedom and *P* value noted<br>*Give P values as exact values whenever suitable.* |
| ☒ | ☐ | For Bayesian analysis, information on the choice of priors and Markov chain Monte Carlo settings |
| ☒ | ☐ | For hierarchical and complex designs, identification of the appropriate level for tests and full reporting of outcomes |
| ☒ | ☐ | Estimates of effect sizes (e.g. Cohen's *d*, Pearson's *r*), indicating how they were calculated |

*Our web collection on statistics for biologists contains articles on many of the points above.*

## Software and code

Policy information about availability of computer code

| | |
|---|---|
| Data collection | 1) www.kmplot.com. - online tool used for Data collection/analysis for Kaplan-Meyer-Survival analysis<br>2) ViiA 7 Real-Time PCR System + Application software version1.2 (Applied Biosystems) - for qPCR data acquisition<br>3) Vectra 3.0 Automated Quantitative Pathology Imaging System (PerkinElmer, Cat# CLS142338) + PhenochartTM v1.0.4 software (PerkinElmer) - acquisition+presentation of immunofluorescence images<br>4) Aperio ImageScope v11.2.0.780 software - used for image acquisition of immunhistochemically stained sections<br>5) BD FACS Canto II + BD FACSDiva V8.0 and BD FACS Aria III + BD FACSDiva V6.0 (BD Biosciences) - used for flow cytometric data acquisition<br>6) Odyssey infrared imaging system + Application software version 3.0 (LI-COR Biosciences) - used to image immunoblotted membranes |
| Data analysis | 1) Fiji  (ImageJ 2.0.0, https://Fiji.sc), Spectrum 11.2.0.780 (Aperio Technologies) and Metamorph (Molecular Devices), - used for quantification of images of immunohistochemically stained sections<br>2) FloJo 10.5.3 (FlowJo, LLC) - used for flow cytometric data analysis<br>3) Prism 7.0c (GraphPad Software, Inc.) - used to generated graphs and perform statistics |

For manuscripts utilizing custom algorithms or software that are central to the research but not yet described in published literature, software must be made available to editors/reviewers. We strongly encourage code deposition in a community repository (e.g. GitHub). See the Nature Research guidelines for submitting code & software for further information.

## Data

Policy information about availability of data

All manuscripts must include a data availability statement. This statement should provide the following information, where applicable:

- Accession codes, unique identifiers, or web links for publicly available datasets
- A list of figures that have associated raw data
- A description of any restrictions on data availability

All relevant data are available from the corresponding author upon reasonable request.

# Field-specific reporting

Please select the one below that is the best fit for your research. If you are not sure, read the appropriate sections before making your selection.

☒ Life sciences ☐ Behavioural & social sciences ☐ Ecological, evolutionary & environmental sciences

For a reference copy of the document with all sections, see nature.com/documents/nr-reporting-summary-flat.pdf

# Life sciences study design

All studies must disclose on these points even when the disclosure is negative.

| | |
|---|---|
| Sample size | G*Power3 was used to calculate minimal required sample size for animal studies based on a difference between means of 40% and a SD of 20% (unpaired, two-tailed t-test; α=0.05, Power=0.95) (p.25, Data acquisition) |
| Data exclusions | No mice or data were excluded after commencement of the data quantification. In a few cases, tissue slides were excluded from downstream analysis due to strong background staining or absence of tumor tissue. These slides were excluded from analysis before commencement of quantification and without knowledge of genotypes/treatment details (aka blinded).(p.27/28, Data acquisition) |
| Replication | All data presented has been replicated. Most graphs display the collective data from several independent experiments and the number of replicate experiments included are given in the figure legends. |
| Randomization | Genetic mouse experiment: Different genotypes from same litter were analysed.<br>Treatment Experiments: All treatment groups were equally distributed within each mouse litter (p.27/28, Data acquisition) |
| Blinding | During assessment of tumor masses, tumor numbers and quantification of IHC stainings, assessor did not know the genotypes or tratement the mice or samples received. (p.27/28, Data acquisition) |

# Reporting for specific materials, systems and methods

We require information from authors about some types of materials, experimental systems and methods used in many studies. Here, indicate whether each material, system or method listed is relevant to your study. If you are not sure if a list item applies to your research, read the appropriate section before selecting a response.

### Materials & experimental systems

| n/a | Involved in the study |
|---|---|
| ☐ | ☒ Antibodies |
| ☒ | ☐ Eukaryotic cell lines |
| ☒ | ☐ Palaeontology |
| ☐ | ☒ Animals and other organisms |
| ☒ | ☐ Human research participants |
| ☒ | ☐ Clinical data |

### Methods

| n/a | Involved in the study |
|---|---|
| ☐ | ☒ ChIP-seq |
| ☐ | ☒ Flow cytometry |
| ☒ | ☐ MRI-based neuroimaging |

## Antibodies

| | |
|---|---|
| Antibodies used | 1) WB antibodies: Used primary antibodies were goat anti-mouse IL-33 (R&D Systems, Cat# AF3626, RRID:AB_884269) and rabbit anti-mouse Gapdh (Sigma-Aldrich, Cat# G9545, RRID:AB_796208). Secondary antibodies used were fluorescent conjugated secondary antibodies IRDye 680 donkey anti-goat ((LI-COR Biosciences, Cat# 926-68074, RRID:AB_10956736) and IRDye 800 goat anti-rabbit ((LI-COR Biosciences, Cat# 926-32211, RRID:AB_621843).<br><br>2) Flow Cytometry antibodies: The following fluorochrome-conjugated antibodies were used for flow cytometric cell sorting and analysis: CD16/CD32 (1/100 dilution, clone 93, Cat# 14-0161-86), EpCAM-FITC (1/400 dilution, clone 9CA ,Cat# 11-5791-82), Ly6c-eF450 (1/300 dilution, HK1.4, Cat# 48-5932-82), F4/80-PE-Cy7 (1/400 dilution, BM8, Cat# 25-4801-82), St2-PerCP-eFluor710 (1:200 dilution, clone RMST2-2, Cat# 46-9335-82), Foxp3-PE (1/200 diltuion, clone FJK-165, Cat# 12-5773-82), Gata3- |

PE (1/200 dilution, clone TWAJ, Cat# 12-9966-42), FceR1-PE-Cy7 (1/300, clone 36951, Cat# 25-5898-82), CD3-PE-Cy7 (1/1000, 145-2C11, Cat# 25-0031-82) all from Ebioscience; CD11b-PE (1/400 dilution, M1/70, Cat# 553311), Ly6g (1/300 dilution, 1A8, Cat# 560602) from BD Pharmingen and CD45.2-A700 (1/400 dilution, clone S450-15-2), CD4 (1/50 dilution, clone GK 1.5), CD11b-PB (1/400 dilution, M1/70), c-Kit-APC (1/200, ACK-2) from WEHI monoclonal antibody facility.

3) Immunohistochemistry antibodies: Primary antibodies used: rabbit anti-mouse CD31 (Abcam, Cat# 28364, RRID: AB_726362), rat anti-mouse F4/80 (Abcam, Cat# 6640, RRID: AB_1140040), rat anti-mouse B220 (BD Biosciences, Cat# 550286, RRID:AB_393581), rabbit anti-mouse CD3 (Abcam, Cat# ab5690, RRID:AB_305055). Secondary antibodies were Biotin-labeled secondary antibodies from the Avidin Biotin Complex ABC-kit (Vector Laboratories).

4) Immunofluorescence antibodies: goat anti-mouse IL-33 (R&D Systems, Cat# AF3626, RRID:AB_884269) and secondary antibody AlexaFluor 568-conjugated donkey anti-goat antibody (Molecular Probes Cat# A-11057, RRID:AB_142581).

Validation

All antibodies have been used according to manufacturer's instructions. For details of verification , relevant citations or further information see the manufacturer's websites.

# Animals and other organisms

Policy information about studies involving animals; ARRIVE guidelines recommended for reporting animal research

Laboratory animals

This study involved mice. All strains are described in the material and methods section, both female and male mice were involved. The age of the mice in an experiment is either stated in the methods section and/or in the figure/ figure legend for each strain and experiment.

Wild animals

This study did not involve wild animals.

Field-collected samples

This study did not involve samples collected from the field.

Ethics oversight

All animal studies were conducted in accordance with the “Australian code for the care and use of animals for scientific purposes” and were approved by the Animal Ethics Committee of the Ludwig Institute for Cancer Research, the Walter and Eliza Hall Institute of Medical Research, or Austin Health.

Note that full information on the approval of the study protocol must also be provided in the manuscript.

# ChIP-seq

## Data deposition

☒ Confirm that both raw and final processed data have been deposited in a public database such as GEO.

☒ Confirm that you have deposited or provided access to graph files (e.g. BED files) for the called peaks.

Data access links
*May remain private before publication.*

https://www.ncbi.nlm.nih.gov/geo/query/acc.cgi?acc=GSE48285

Files in database submission

GSE48285_RAW.tar
GSM1174211_Control_MACS_Peaks.bed.gz
GSM1174211_Control_Peak_Information_MACS_Output_.xls.gz
GGSM1174212_IL6_MACS_Peaks.bed.gz
GSM1174212_IL6_Peak_Information_MACS_Output_.xls.gz
GSM1174213_IL11_MACS_Peaks.bed.gz
GSM1174213_IL11_Peak_Information_MACS_Output_.xls.gz

Genome browser session
(e.g. UCSC)

no longer applicable

## Methodology

Replicates

Stomach tumor from 3 mice were dissected and pooled for processing for each sample analysed. No replicate experiment was performed.

Sequencing depth

35bp, single-end sequencing was performed on a Ilumina genome analyzer II for each sample

Antibodies

rabbit anti-mouse Stat3 antibody (Santa Cruz Biotechnologies, cat# SC-482X, clone C20, RRID:AB_632440).

Peak calling parameters

Peak calling was performed using MACS software (Zheng et al., 2008) with a fold-enrichment ratio (mfold) of 12 fold and a standard threshold probability (p-value) of P≤10-5.

Data quality

Base-calling: Base-calling was performed using Illumina software. Reads that have poor sequence quality and/or are made of homopolymers were removed from the datasets.
Read alignment: After filtering of poor quality sequences, ChIP-Seq reads were aligned to the UCSC mm9 genome assembly using Bowtie software.

| | |
|---|---|
| | File conversion: Conversion of Bowtie output files to .bed and .wig files was performed using Perl.<br>Peak calling: Peak calling was performed using MACS software (Zheng et al., 2008) with a fold-enrichment ratio (mfold) of 12 fold and a standard threshold probability (p-value) of P≤10-5.<br>Retrieval of relevant peak and associated gene/microRNA information was performed using R programming language for statistical computing (Version 2.12.1).<br>The three processed data files "Control_Peak_Information_MACS_Output.xls", "IL6_Peak_Information_MACS_Output.xls" and "IL11_Peak_Information _MACS_Output.xls" are the original output files of the MACS software and contain the following information about significant Stat3-associated peaks: (I) exact peak location (chromosome number and the start and end nucleotide positions), (II) peak width/length (expressed in base pairs), (III) peak summit (the exact nucleotide position in each peak at which the highest number of tags were counted), (IV) reads/tags per peak (total number of sequence tags counted at each enriched region ), (V) P-value and (VI) fold enrichment of each peak. |
| Software | Base-calling: Base-calling was performed using Illumina software.<br>Read alignment: ChIP-Seq reads were aligned to the UCSC mm9 genome assembly using Bowtie software.<br>File conversion: Conversion of Bowtie output files to .bed and .wig files was performed using Perl.<br>Peak calling: Peak calling was performed using MACS software (Zheng et al., 2008)<br>Retrieval of relevant peak and associated gene/microRNA information was performed using R programming language for statistical computing (Version 2.12.1). |

# Flow Cytometry

## Plots

Confirm that:

☒ The axis labels state the marker and fluorochrome used (e.g. CD4-FITC).

☒ The axis scales are clearly visible. Include numbers along axes only for bottom left plot of group (a 'group' is an analysis of identical markers).

☒ All plots are contour plots with outliers or pseudocolor plots.

☒ A numerical value for number of cells or percentage (with statistics) is provided.

## Methodology

| | |
|---|---|
| Sample preparation | Glandular stomachs or antral tumors were dissected and enzymatically digested as previously described manuscript Ref 76. |
| Instrument | FACS analyzer: FACS Canto II (BD Bioscience), FACS sorting: FACS Aria III (BD Biosicence) |
| Software | FACS analyzer: BD FACSDiva V8.0<br>FACS sorting: BD FACSDiva V6.0 |
| Cell population abundance | Deails regaring the abundance of the populations and the purity are contained within the relevant figures/ figure legends. |
| Gating strategy | For all analysis gating was set for: cells (gate FSC-A - SSC-A), doublet exclusion (gate: FSC-W - FSC-H), alive cells (Propidium Iodide -). Mast cells sorting: From CD45+EpCam- cell population the CD11b- cells were selected, then finally the c-Kit+FceRI+ population represents the mast cells. Macrophage sorting: From CD45+EpCam- cells, the F4/80HighCD11b+ population was selected and back-gated to confirm that the selected macrophage population was Ly6C-Ly6G-. |

☒ Tick this box to confirm that a figure exemplifying the gating strategy is provided in the Supplementary Information.
